# Supplementary material for: Delay discounting and family history of psychopathology in children ages 9–11
Source: Sci Rep. 2023 Dec 11;13:21977. doi: 10.1038/s41598-023-49148-4 (PMC10713649; doi:10.1038/s41598-023-49148-4)
Supplement: Supplementary file 1 — Supplementary Information. [file 41598_2023_49148_MOESM1_ESM.docx]

**Figure S1. Correlations between Family Pattern Density Scores in the Full Sample**


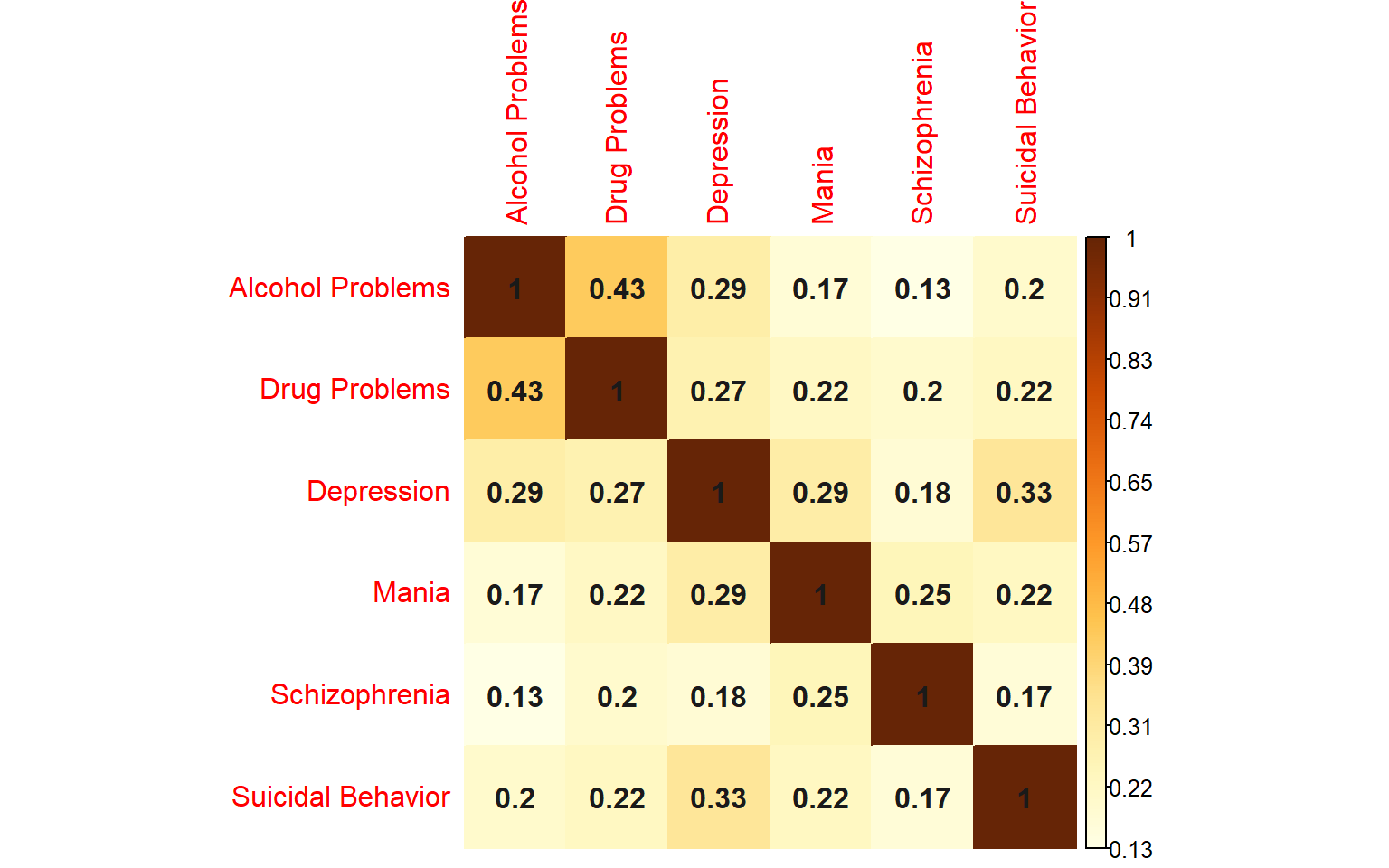


Spearman’s correlations between family pattern density scores in the full sample. All correlations were significant (p’s < 0.001).

**Figure S2. Correlations between Family Pattern Density Scores and Delay Discounting Measures in the Quality Control Sample**

**
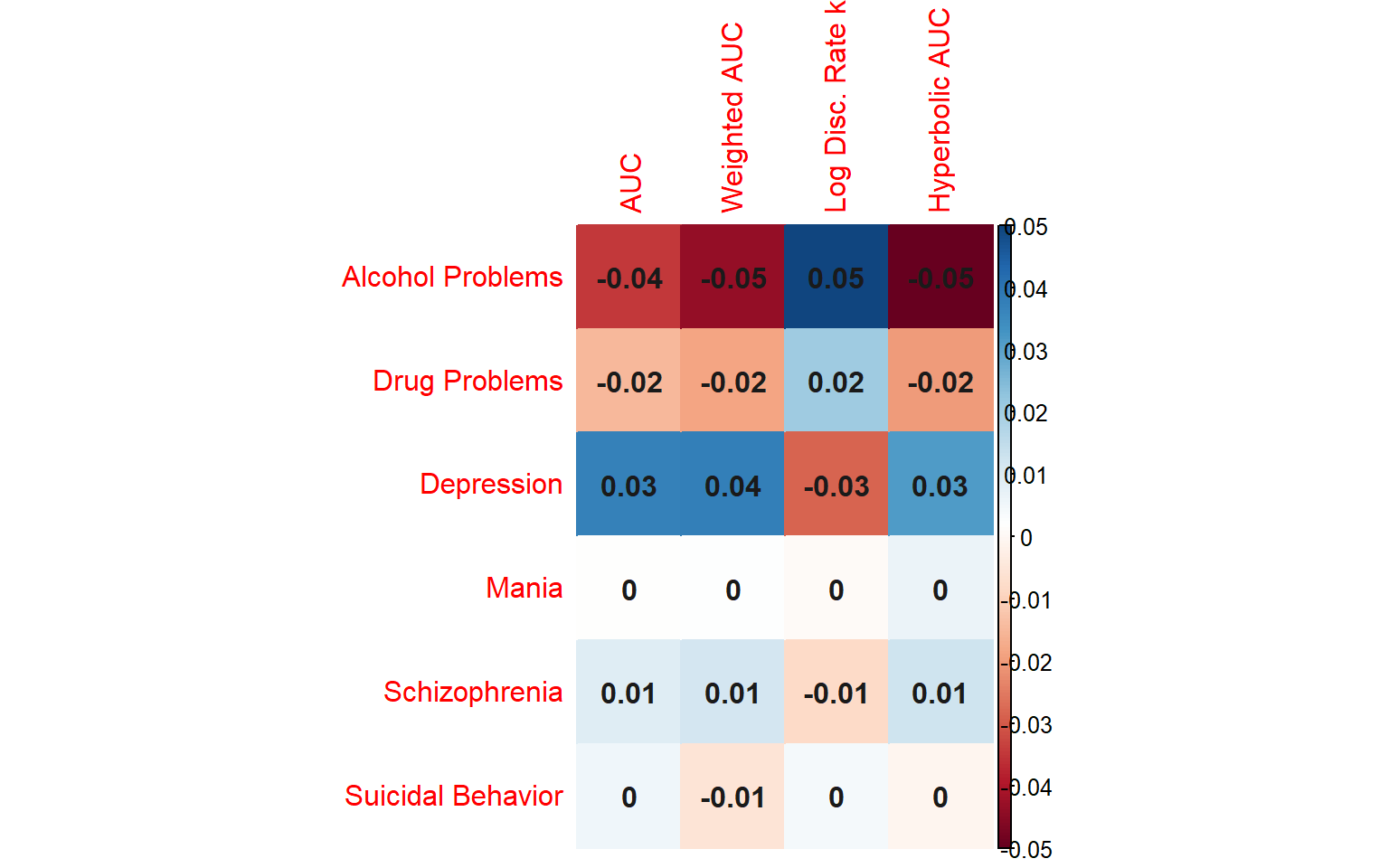
**

Spearman’s correlations between family pattern density scores and delay discounting measures in the sample that met data quality criteria for the delay discounting task (N=4364).

**Figure S3. Correlations between Family History Density Scores and Delay Discounting Measures in the Full Sample**


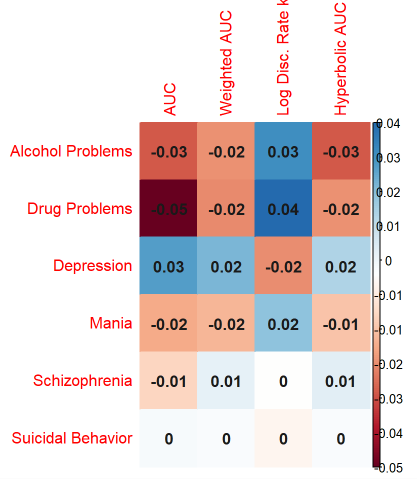


Spearman’s correlations between family history density scores and delay discounting measures in the full sample.

**Figure S4. Correlations between Family Patterns Analysis Scores and Delay Discounting Measures in the Full Sample**


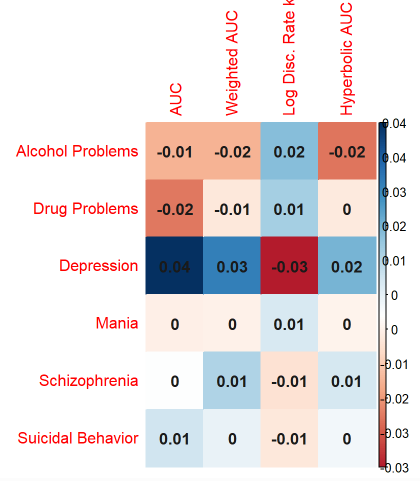


Spearman’s correlations between family patterns analysis scores and delay discounting measures in the full sample.

**Figure S5. Association between Family History of Alcohol Problems and Delay Discounting Behavior in the Full Sample with Sociodemographic Factors Included**

**
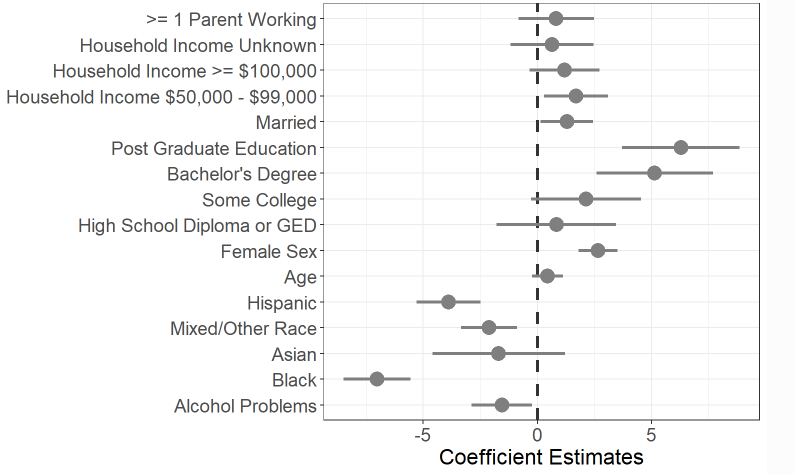
**

Full mixed effects model examining the association between family pattern density of alcohol problems and delay discounting behavior in the full sample. The model did not find a significant association between family history of alcohol problems and delay discounting behavior when adjusted for socioeconomic and demographic variables after Bonferroni correction. Error bars represent 95% confidence intervals for unstandardized coefficient estimates. In this figure, the area under the indifference point versus delay curve is the dependent variable, so lower coefficient values indicate greater delay discounting.

**Figure S6. Association between Family History of Drug Problems and Delay Discounting Behavior in the Full Sample with Sociodemographic Factors Included**

**
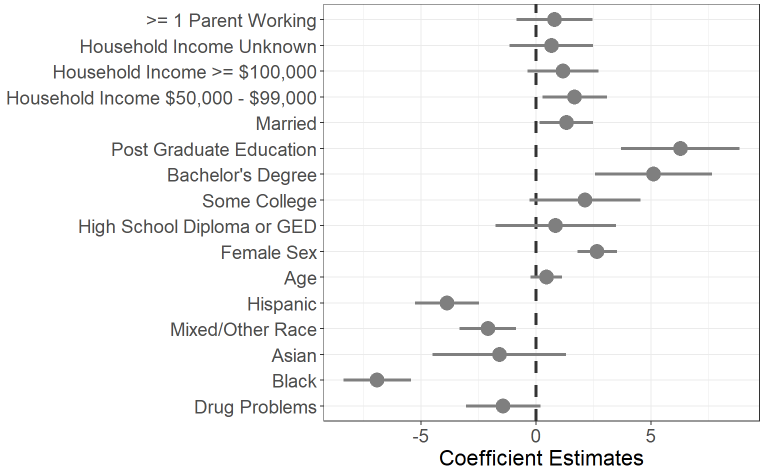
**

Full mixed effects model examining the association between family pattern density of drug problems and delay discounting behavior in the full sample. The model did not find a significant association between family history of drug problems and delay discounting behavior when adjusted for socioeconomic and demographic variables after Bonferroni correction. Error bars represent 95% confidence intervals for unstandardized coefficient estimates. Area under the indifference point versus delay curve is the dependent variable, so lower coefficient values indicate greater delay discounting.

**Figure S7. Association between Family History of Depression and Delay Discounting Behavior in the Full Sample with Sociodemographic Factors Included**

**
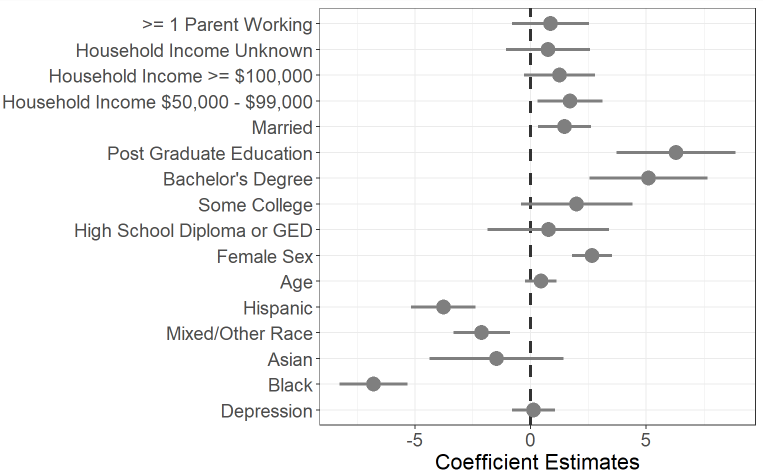
**

Full mixed effects model examining the association between family pattern density of depression and delay discounting behavior in the full sample. The model did not find a significant association between family history of depression and delay discounting behavior when adjusted for socioeconomic and demographic variables after Bonferroni correction. Error bars represent 95% confidence intervals for unstandardized coefficient estimates. Area under the indifference point versus delay curve is the dependent variable, so lower coefficient values indicate greater delay discounting.

**Figure S8. Association between Family History of Mania and Delay Discounting Behavior in the Full Sample with Sociodemographic Factors Included**

**
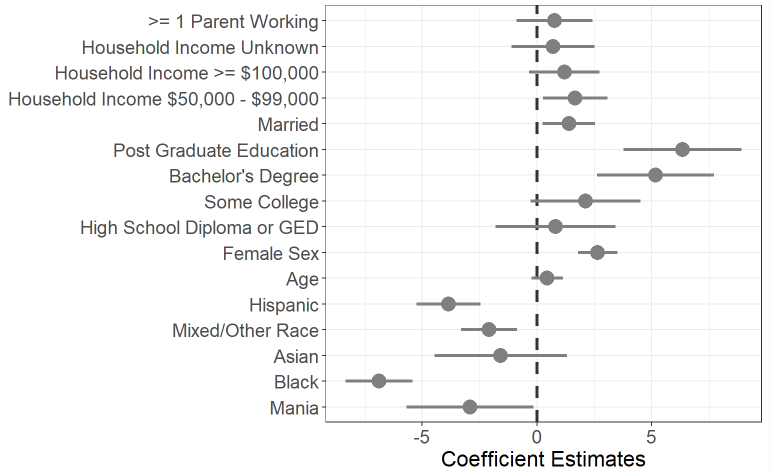
**

Full mixed effects model examining the association between family pattern density of mania and delay discounting behavior in the full sample. The model did not find a significant association between family history of mania and delay discounting behavior when adjusted for socioeconomic and demographic variables after Bonferroni correction. Error bars represent 95% confidence intervals for unstandardized coefficient estimates. Area under the indifference point versus delay curve is the dependent variable, so lower coefficient values indicate greater delay discounting.

**Figure S9. Association between Family History of Schizophrenia and Delay Discounting Behavior in the Full Sample with Sociodemographic Factors Included**

**
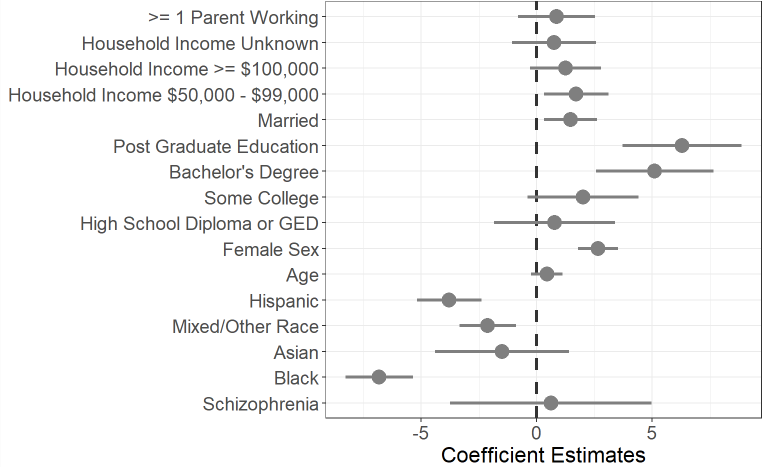
**

Full mixed effects model examining the association between family pattern density of schizophrenia and delay discounting behavior in the full sample. The model did not find a significant association between family history of schizophrenia and delay discounting behavior when adjusted for socioeconomic and demographic variables after Bonferroni correction. Error bars represent 95% confidence intervals for unstandardized coefficient estimates. Area under the indifference point versus delay curve is the dependent variable, so lower coefficient values indicate greater delay discounting.

**Figure S10. Association between Family History of Suicidal Behavior and Delay Discounting Behavior in the Full Sample with Sociodemographic Factors Included**

**
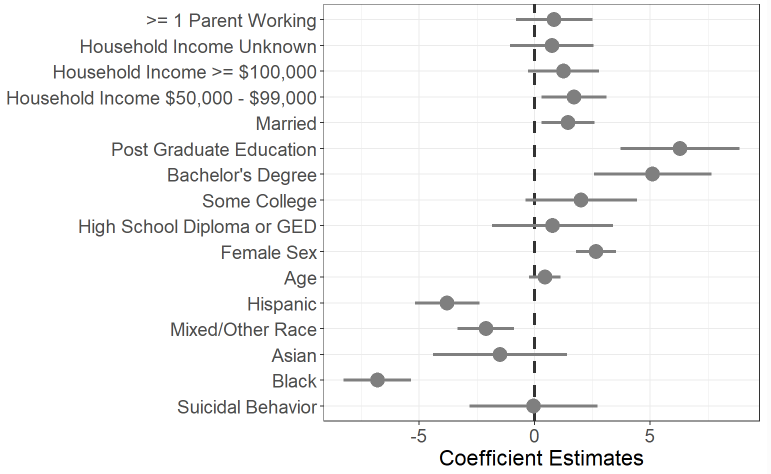
**

Full mixed effects model examining the association between family pattern density of suicidal behavior and delay discounting behavior in the full sample. The model did not find a significant association between family history of suicidal behavior and delay discounting behavior when adjusted for socioeconomic and demographic variables after Bonferroni correction. Error bars represent 95% confidence intervals for unstandardized coefficient estimates. Area under the indifference point versus delay curve is the dependent variable, so lower coefficient values indicate greater delay discounting.

**Figure S11. Associations between Family History of Psychopathology and Delay Discounting Behavior in the Quality Control Sample with Sociodemographic Factors Included**

**
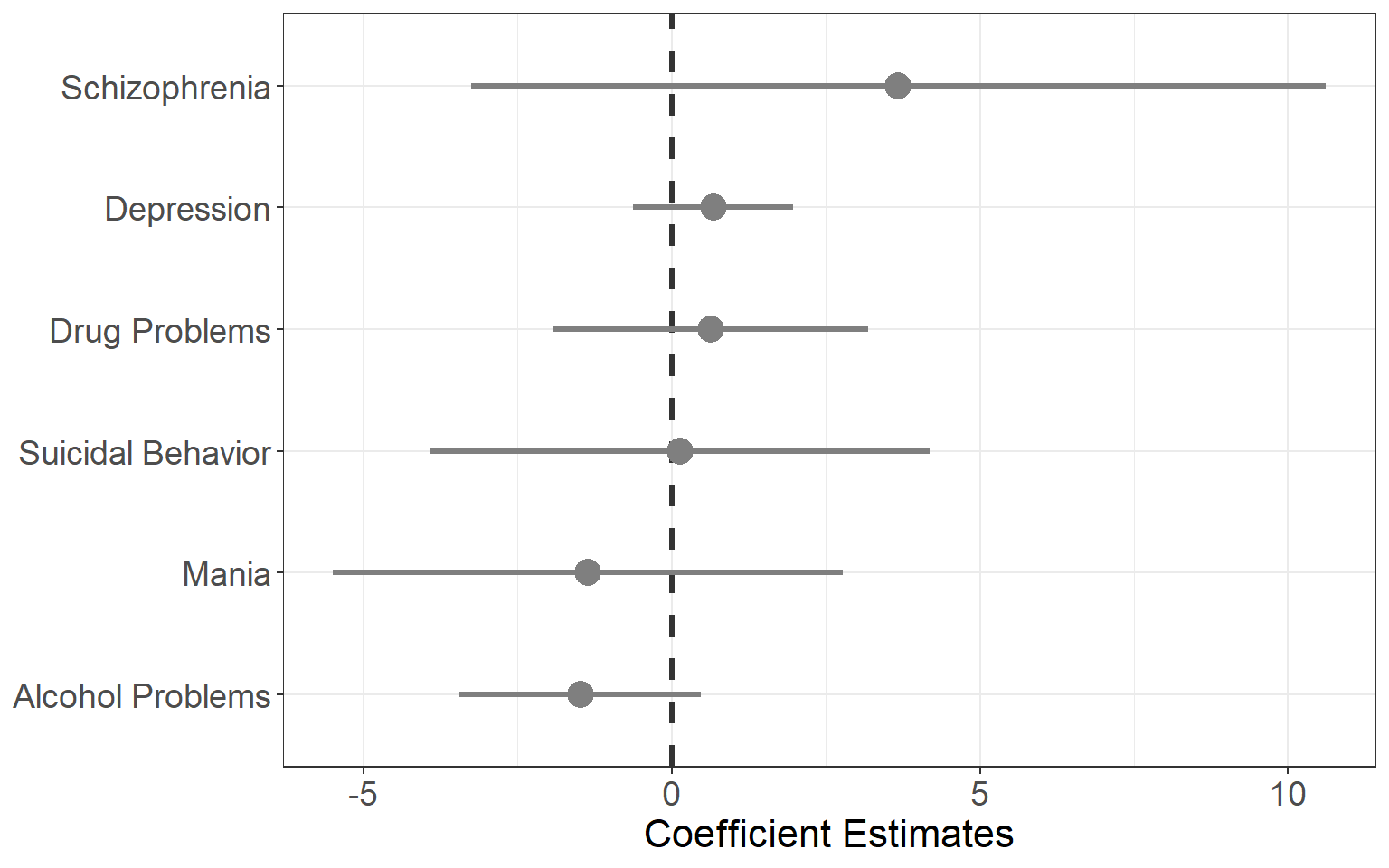
**

Coefficient estimates from adjusted mixed effects models examining the association between family patterns density scores and delay discounting behavior in the sample that met data quality criteria for the delay discounting task (N=4364). Mixed effects models did not find any significant associations between family history of psychiatric disorders and delay discounting behavior when adjusted for socioeconomic and demographic variables. Error bars represent 95% confidence intervals for unstandardized coefficient estimates. Area under the indifference point versus delay curve is the dependent variable, so lower coefficient values indicate greater delay discounting.

**Figure S12. Association between Family History of Alcohol Problems and Delay Discounting Behavior in the Quality Control Sample with Sociodemographic Factors Included**

**
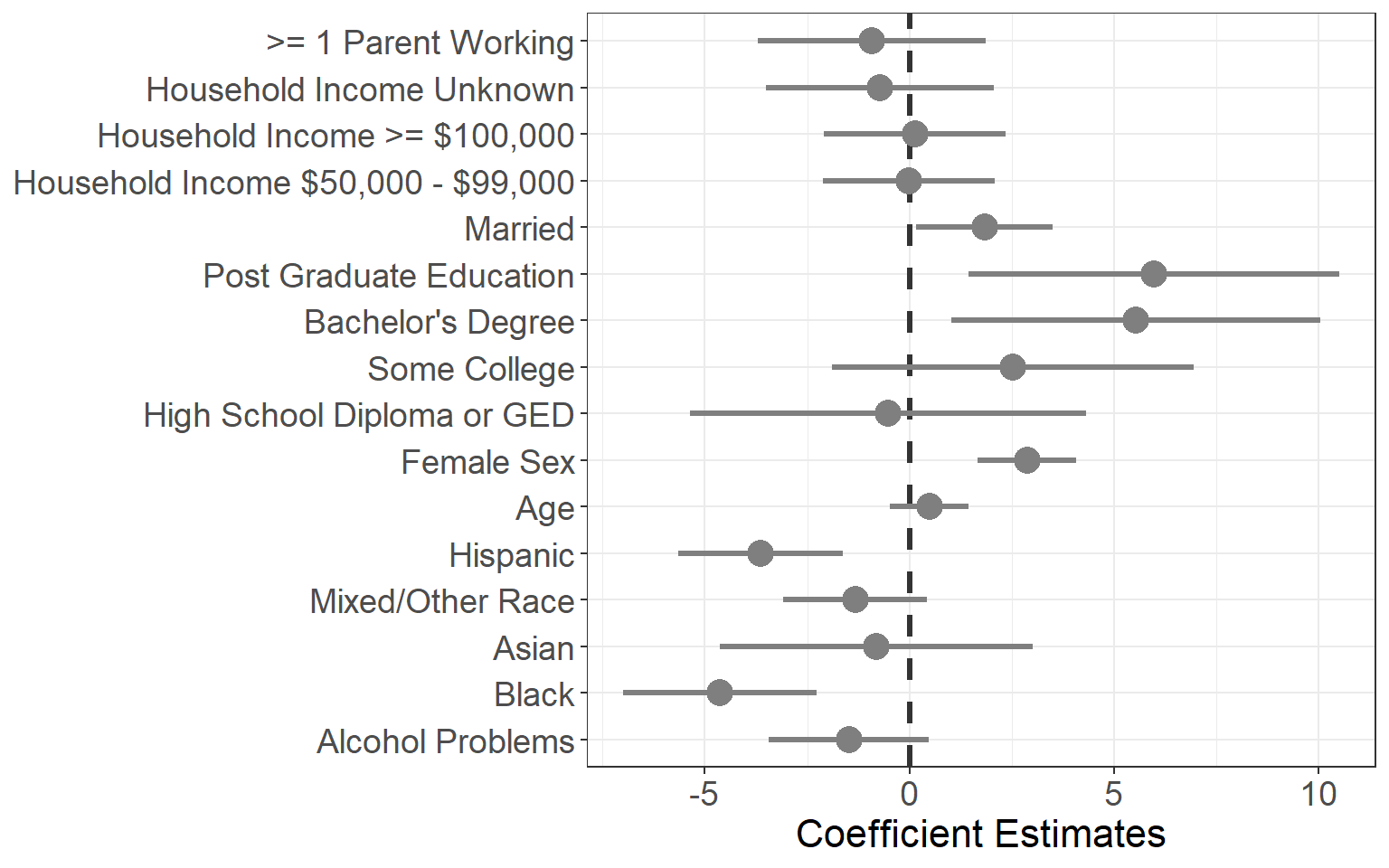
**

Full mixed effects model examining the association between family pattern density of alcohol problems and delay discounting behavior in the sample that met data quality criteria for the delay discounting task (N=4364). The model did not find a significant association between family history of alcohol problems and delay discounting behavior when adjusted for socioeconomic and demographic variables. Error bars represent 95% confidence intervals for unstandardized coefficient estimates. Area under the indifference point versus delay curve is the dependent variable, so lower coefficient values indicate greater delay discounting.

**Figure S13. Association between Family History of Drug Problems and Delay Discounting Behavior in the Quality Control Sample with Sociodemographic Factors Included**

**
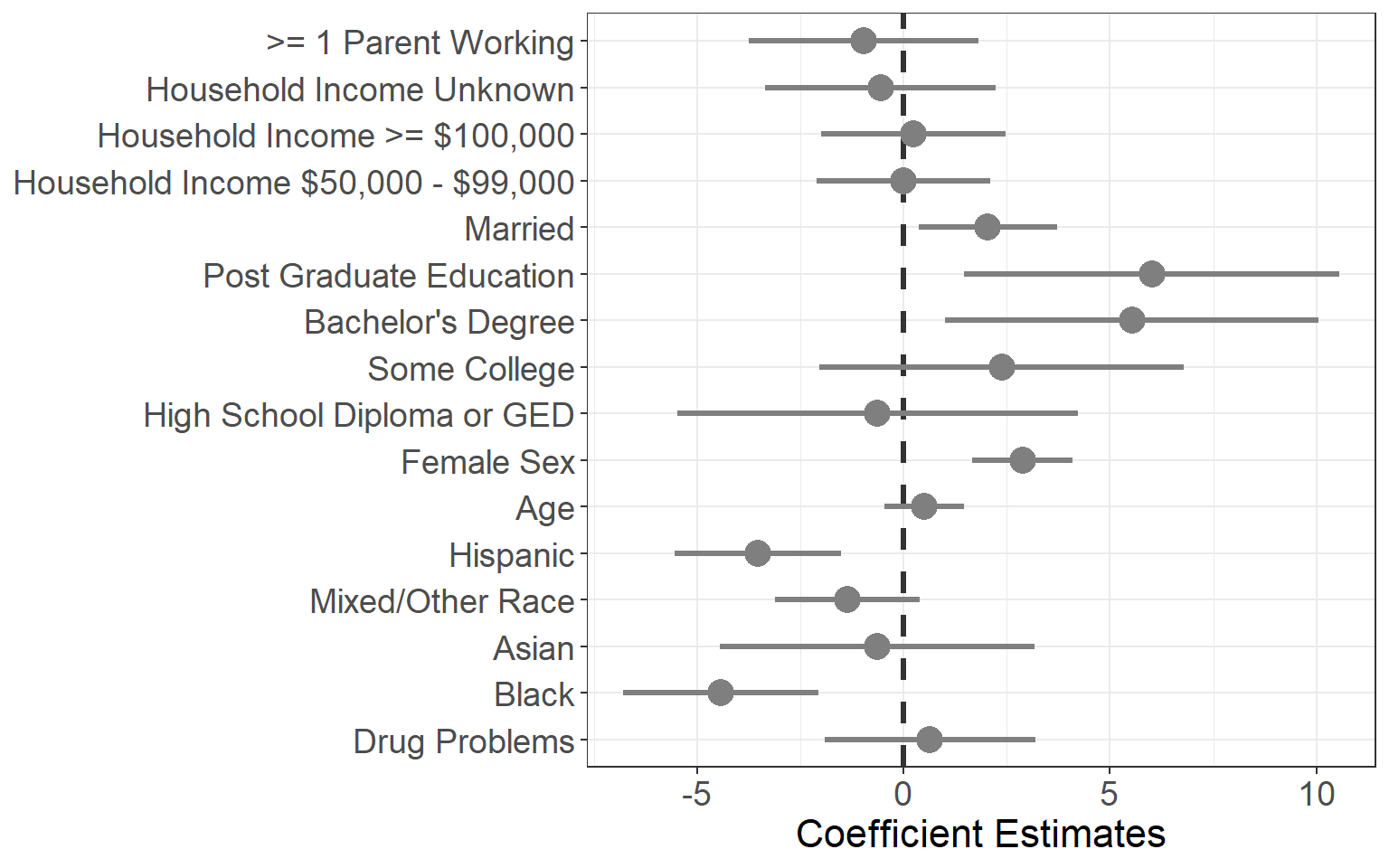
**

Full mixed effects model examining the association between family pattern density of drug problems and delay discounting behavior in the sample that met data quality criteria for the delay discounting task (N=4364). The model did not find a significant association between family history of drug problems and delay discounting behavior when adjusted for socioeconomic and demographic variables. Error bars represent 95% confidence intervals for unstandardized coefficient estimates. Area under the indifference point versus delay curve is the dependent variable, so lower coefficient values indicate greater delay discounting.

**Figure S14. Association between Family History of Depression and Delay Discounting Behavior in the Quality Control Sample with Sociodemographic Factors Included**

**
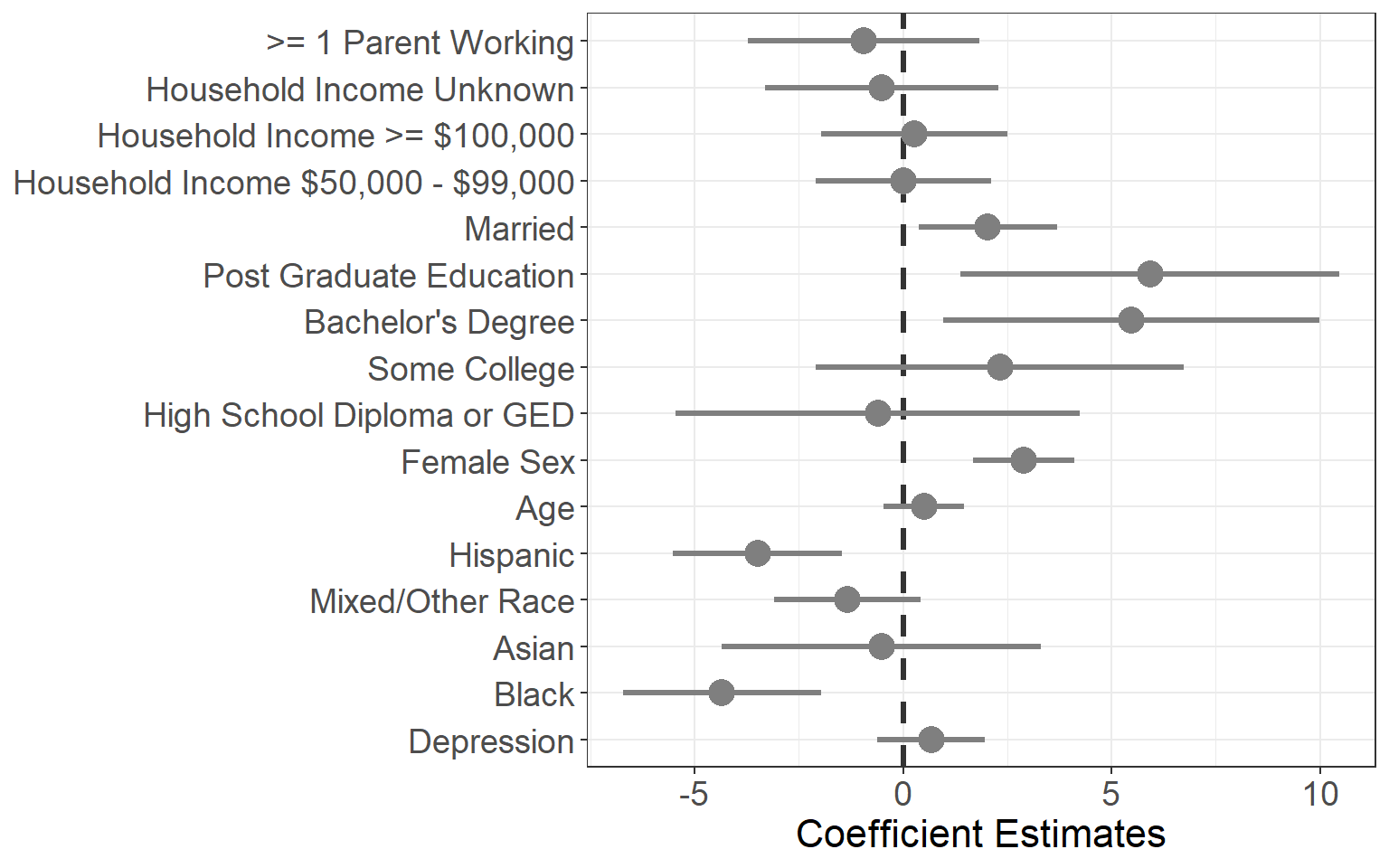
**

Full mixed effects model examining the association between family pattern density of depression and delay discounting behavior in the sample that met data quality criteria for the delay discounting task (N=4364). The model did not find a significant association between family history of depression and delay discounting behavior when adjusted for socioeconomic and demographic variables. Error bars represent 95% confidence intervals for unstandardized coefficient estimates. Area under the indifference point versus delay curve is the dependent variable, so lower coefficient values indicate greater delay discounting.

**Figure S15. Association between Family History of Mania and Delay Discounting Behavior in the Quality Control Sample with Sociodemographic Factors Included**

**
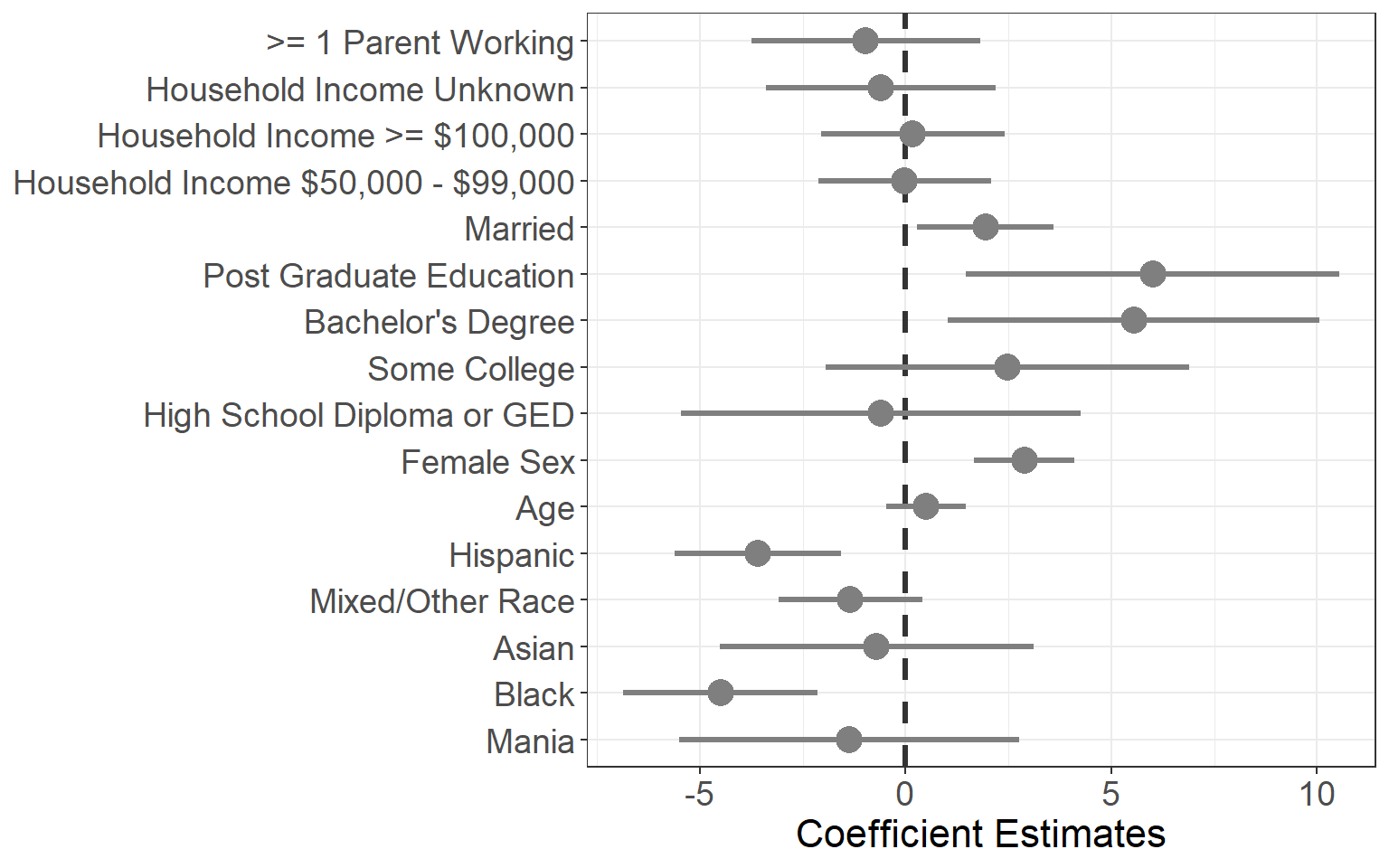
**

Full mixed effects model examining the association between family pattern density of mania and delay discounting behavior in the sample that met data quality criteria for the delay discounting task (N=4364). The model did not find a significant association between family history of mania and delay discounting behavior when adjusted for socioeconomic and demographic variables. Error bars represent 95% confidence intervals for unstandardized coefficient estimates. Area under the indifference point versus delay curve is the dependent variable, so lower coefficient values indicate greater delay discounting.

**Figure S16. Association between Family History of Schizophrenia and Delay Discounting Behavior in the Quality Control Sample with Sociodemographic Factors Included**

**
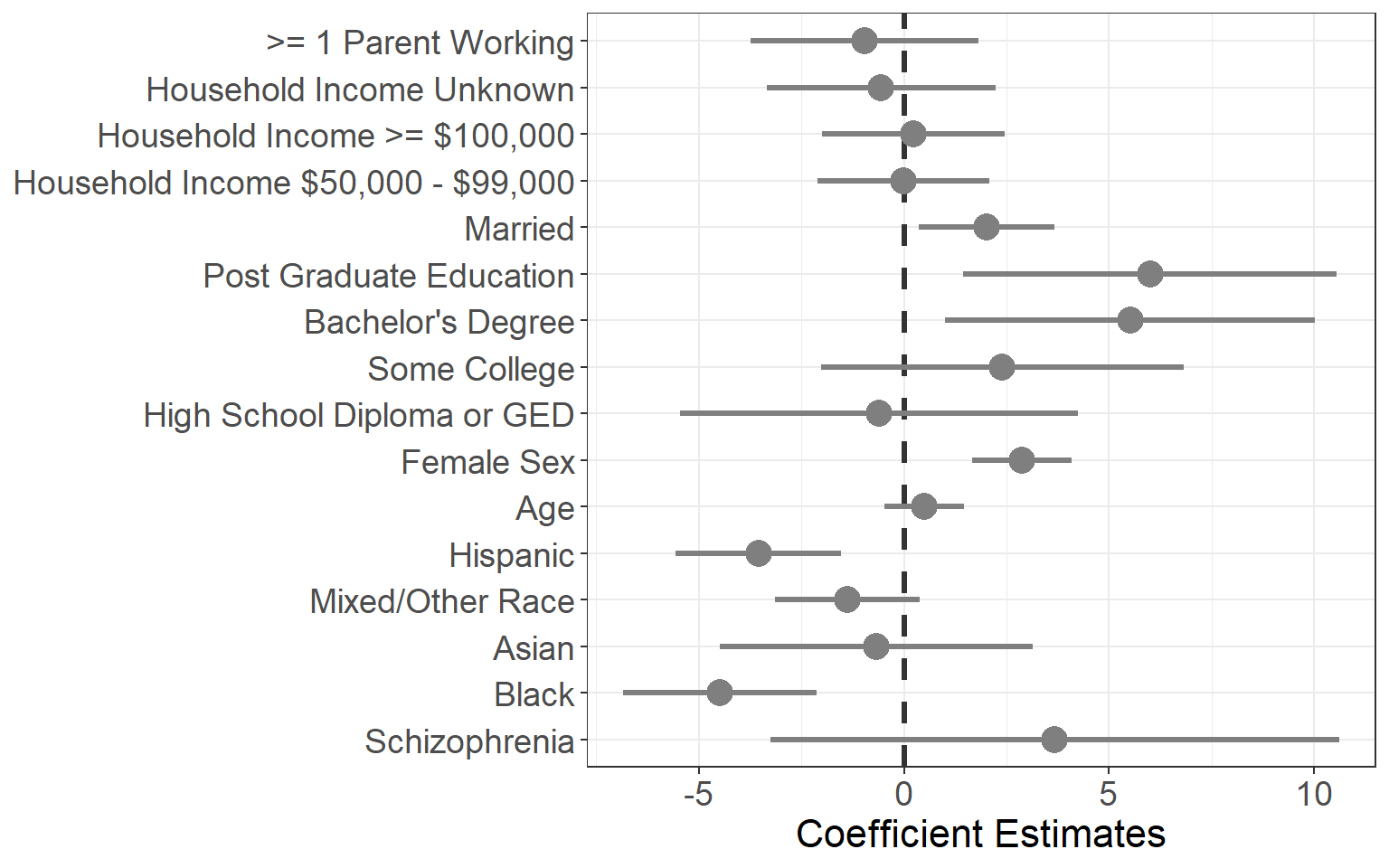
**

Full mixed effects model examining the association between family pattern density of schizophrenia and delay discounting behavior in the sample that met data quality criteria for the delay discounting task (N=4364). The model did not find a significant association between family history of schizophrenia and delay discounting behavior when adjusted for socioeconomic and demographic variables. Error bars represent 95% confidence intervals for unstandardized coefficient estimates. Area under the indifference point versus delay curve is the dependent variable, so lower coefficient values indicate greater delay discounting.

**Figure S17. Association between Family History of Suicidal Behavior and Delay Discounting Behavior in the Quality Control Sample with Sociodemographic Factors Included**

**
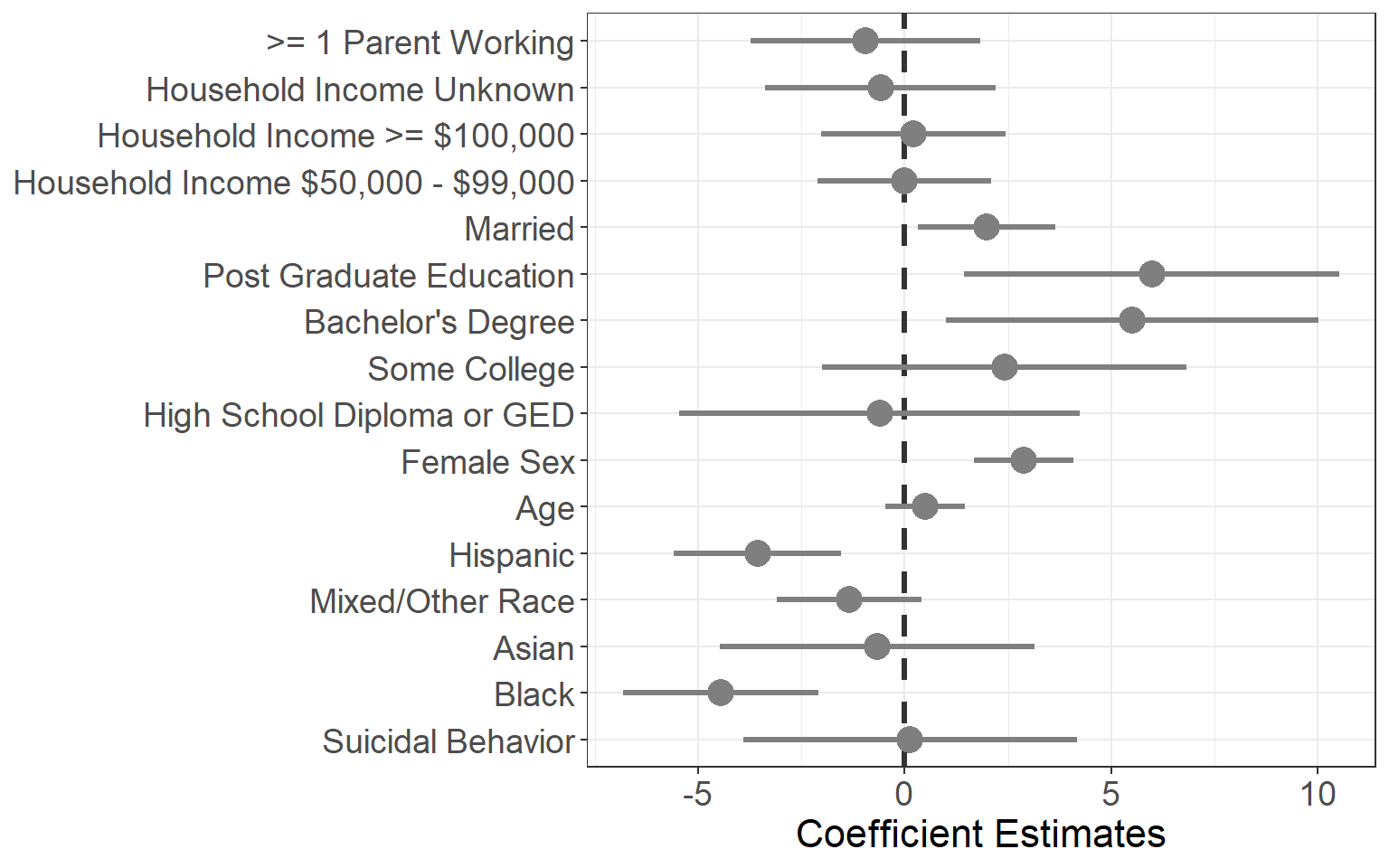
**

Full mixed effects model examining the association between family pattern density of suicidal behavior and delay discounting behavior (area under the curve) in the sample that met data quality criteria for the delay discounting task (N=4364). The model did not find a significant association between family history of suicidal behavior and delay discounting behavior when adjusted for socioeconomic and demographic variables. Error bars represent 95% confidence intervals for unstandardized coefficient estimates. Area under the indifference point versus delay curve is the dependent variable, so lower coefficient values indicate greater delay discounting.

**Figure S18. Associations between Family History Density Scores and Delay Discounting Behavior in the Full Sample with Sociodemographic Factors Included**

**
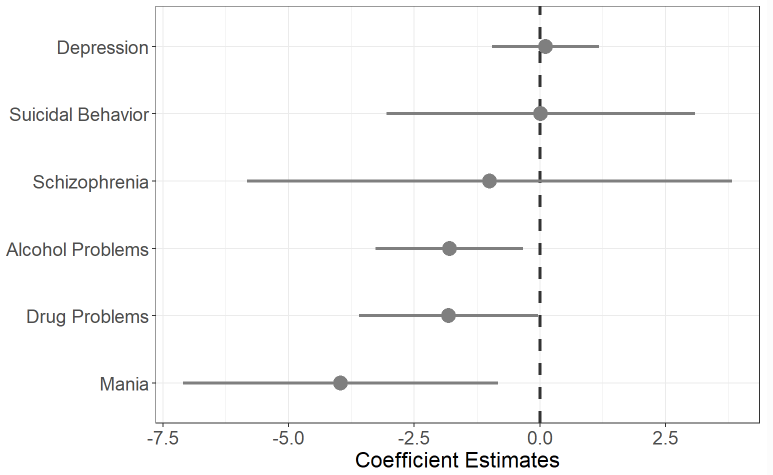
**

Coefficient estimates from adjusted mixed effects models examining the association between family history density scores and delay discounting behavior in the full sample. Mixed effects models did not find any significant associations between family history of psychiatric disorders and delay discounting behavior when adjusted for socioeconomic and demographic variables. Error bars represent 95% confidence intervals for unstandardized coefficient estimates. Although the 95% confidence intervals for alcohol problems and mania do not intersect with zero, these effects are no longer significant after applying a Bonferroni correction to control for multiple comparisons. Area under the indifference point versus delay curve is the dependent variable, so lower coefficient values indicate greater delay discounting.

**Figure S19. Associations between Family Patterns Analysis Scores and Delay Discounting Behavior in the Full Sample with Sociodemographic Factors Included**

**
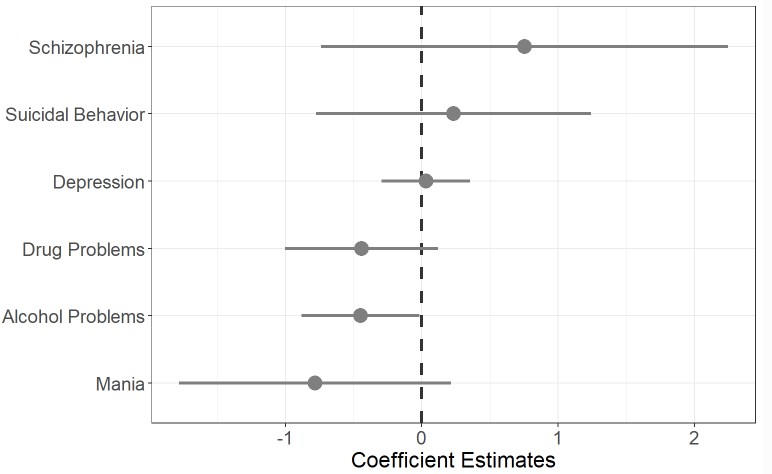
**

Coefficient estimates from adjusted mixed effects models examining the association between family patterns analysis scores and delay discounting behavior in the full sample. Mixed effects models did not find any significant associations between family history of psychiatric disorders and delay discounting behavior when adjusted for socioeconomic and demographic variables. Error bars represent 95% confidence intervals for unstandardized coefficient estimates. Area under the indifference point versus delay curve is the dependent variable, so lower coefficient values indicate greater delay discounting.

**Table S1. Mixed Effects Model Examining the Association between Family History of Alcohol Problems and Delay Discounting Behavior in the Full Sample with Sociodemographic Factors Included**

| Predictors | Estimates | 95% CI | p |
| --- | --- | --- | --- |
| Family History of Alcohol Problems | -1.55 | -2.88, -0.22 | 0.022 |
| **Black** | **-7.02** | **-8.48, -5.55** | **<0.001** |
| Asian | -1.69 | -4.59, 1.21 | 0.253 |
| **Mixed/Other Race** | **-2.10** | **-3.33, -0.88** | **0.001** |
| **Hispanic** | **-3.88** | **-5.28, -2.49** | **<0.001** |
| Age | 0.45 | -0.24, 1.13 | 0.199 |
| **Female** | **2.66** | **1.80, 3.52** | **<0.001** |
| High School Diploma or GED | 0.84 | -1.79, 3.46 | 0.532 |
| Some College | 2.13 | -0.27, 4.54 | 0.082 |
| **Bachelor’s Degree** | **5.14** | **2.60, 7.69** | **<0.001** |
| **Post Graduate Education** | **6.29** | **3.71, 8.86** | **<0.001** |
| Married | 1.30 | 0.14, 2.45 | 0.028 |
| Household Income $50,000 - $99,000 | 1.70 | 0.30, 3.10 | 0.018 |
| Household Income ≥ $100,000 | 1.20 | -0.34, 2.73 | 0.126 |
| Household Income Unknown | 0.65 | -1.16, 2.46 | 0.482 |
| ≥1 Parent Working | 0.83 | -0.83, 2.49 | 0.326 |
| Random Effects |  |  |  |
| σ | 453.57 |  |  |
| τ_00_ Family | 55.19 |  |  |
| τ_00_ Site | 0.74 |  |  |
| ICC | 0.11 |  |  |
| N_Family_ | 8944 |  |  |
| N_Site_ | 22 |  |  |
| Observations | 10788 |  |  |
| Marginal R^2^ | 0.049 |  |  |
| Conditional R^2^ | 0.152 |  |  |
|  |  |  |  |

Full mixed effects model examining the association between family pattern density of alcohol problems and delay discounting behavior (area under the curve) in the full sample. The model did not find a significant association between family history of alcohol problems and delay discounting behavior when adjusted for socioeconomic and demographic variables and applying Bonferroni correction for six comparisons.

**Table S2. Mixed Effects Model Examining the Association between Family History of Drug Problems and Delay Discounting Behavior in the Full Sample with Sociodemographic Factors Included**

| Predictors | Estimates | 95% CI | p |
| --- | --- | --- | --- |
| Family History of Drug Problems | -1.43 | -3.04, 0.19 | 0.084 |
| **Black** | **-6.90** | **-8.37, -5.44** | **<0.001** |
| Asian | -1.60 | -4.49, 1.30 | 0.280 |
| **Mixed/Other Race** | **-2.09** | **-3.32, -0.86** | **0.001** |
| **Hispanic** | **-3.87** | **-5.27, -2.48** | **<0.001** |
| Age | 0.45 | -0.23, 1.13 | 0.197 |
| **Female** | **2.66** | **1.80, 3.52** | **<0.001** |
| High School Diploma or GED | 0.86 | -1.76, 3.48 | 0.521 |
| Some College | 2.13 | -0.28, 4.54 | 0.083 |
| **Bachelor’s Degree** | **5.11** | **2.56, 7.65** | **<0.001** |
| **Post Graduate Education** | **6.28** | **3.70, 8.85** | **<0.001** |
| Married | 1.32 | 0.16, 2.48 | 0.025 |
| Household Income $50,000 - $99,000 | 1.68 | 0.28, 3.09 | 0.019 |
| Household Income ≥ $100,000 | 1.17 | -0.36, 2.71 | 0.134 |
| Household Income Unknown | 0.67 | -1.14, 2.48 | 0.469 |
| ≥ 1 Parent Working | 0.81 | -0.85, 2.47 | 0.340 |
| Random Effects |  |  |  |
| σ | 453.93 |  |  |
| τ_00_ Family | 53.9 |  |  |
| τ_00_ Site | 0.79 |  |  |
| ICC | 0.11 |  |  |
| N_Family_ | 8944 |  |  |
| N_Site_ | 22 |  |  |
| Observations | 10788 |  |  |
| Marginal R^2^ | 0.049 |  |  |
| Conditional R^2^ | 0.151 |  |  |
|  |  |  |  |

Full mixed effects model examining the association between family pattern density of drug problems and delay discounting behavior (area under the curve) in the full sample. The model did not find a significant association between family history of drug problems and delay discounting behavior when adjusted for socioeconomic and demographic variables.

**Table S3. Mixed Effects Model Examining the Association between Family History of Depression and Delay Discounting Behavior in the Full Sample with Sociodemographic Factors Included**

| Predictors | Estimates | 95% CI | p |
| --- | --- | --- | --- |
| Family History of Depression | 0.13 | -0.80, 1.06 | 0.787 |
| **Black** | **-6.78** | **-8.26, -5.31** | **<0.001** |
| Asian | -1.47 | -4.37, 1.43 | 0.322 |
| **Mixed/Other Race** | **-2.11** | **-3.34, -0.88** | **0.001** |
| **Hispanic** | **-3.77** | **-5.16, -2.37** | **<0.001** |
| Age | 0.44 | -0.24, 1.13 | 0.203 |
| **Female Sex** | **2.66** | **1.80, 3.52** | **<0.001** |
| High School Diploma or GED | 0.77 | -1.85, 3.40 | 0.563 |
| Some College | 2.00 | -0.41, 4.40 | 0.104 |
| **Bachelor’s Degree** | **5.09** | **2.55, 7.64** | **<0.001** |
| **Post Graduate Education** | **6.29** | **3.71, 8.87** | **<0.001** |
| Married | 1.47 | 0.32, 2.62 | 0.012 |
| Household Income $50,000 - $99,000 | 1.72 | 0.31, 3.12 | 0.017 |
| Household Income ≥ $100,000 | 1.26 | -0.27, 2.79 | 0.108 |
| Household Income Unknown | 0.76 | -1.06, 2.57 | 0.412 |
| ≥Parent Working | 0.86 | -0.80, 2.52 | 0.309 |
| Random Effects |  |  |  |
| σ | 454.03 |  |  |
| τ_00_ Family | 53.94 |  |  |
| τ_00_ Site | 0.78 |  |  |
| ICC | 0.11 |  |  |
| N_Family_ | 8944 |  |  |
| N_Site_ | 22 |  |  |
| Observations | 10788 |  |  |
| Marginal R^2^ | 0.049 |  |  |
| Conditional R^2^ | 0.151 |  |  |
|  |  |  |  |

Full mixed effects model examining the association between family pattern density of depression and delay discounting behavior (area under the curve) in the full sample. The model did not find a significant association between family history of depression and delay discounting behavior when adjusted for socioeconomic and demographic variables.

**Table S4. Mixed Effects Model Examining the Association between Family History of Mania and Delay Discounting Behavior in the Full Sample with Sociodemographic Factors Included**

| Predictors | Estimates | 95% CI | p |
| --- | --- | --- | --- |
| Family History of Mania | -2.91 | -5.69, -0.14 | 0.040 |
| **Black** | **-6.88** | **-8.35, -5.42** | **<0.001** |
| Asian | -1.58 | -4.47, 1.32 | 0.285 |
| **Mixed/Other Race** | **-2.09** | **-3.31, -0.86** | **0.001** |
| **Hispanic** | **-3.86** | **-5.26, -2.47** | **<0.001** |
| Age | 0.45 | -0.24, 1.13 | 0.201 |
| **Female** | **2.64** | **1.79, 3.50** | **<0.001** |
| High School Diploma or GED | 0.81 | -1.81, 3.43 | 0.544 |
| Some College | 2.12 | -0.29, 4.52 | 0.085 |
| **Bachelor’s Degree** | **5.17** | **2.62, 7.71** | **<0.001** |
| **Post Graduate Education** | **6.35** | **3.78, 8.92** | **<0.001** |
| Married | 1.39 | 0.24, 2.54 | 0.018 |
| Household Income $50,000 - $99,000 | 1.66 | 0.26, 3.07 | 0.020 |
| Household Income ≥ $100,000 | 1.19 | -0.34, 2.73 | 0.127 |
| Household Income Unknown | 0.69 | -1.12, 2.51 | 0.452 |
| ≥ 1 Parent Working | 0.77 | -0.89, 2.43 | 0.365 |
| Random Effects |  |  |  |
| σ | 454.09 |  |  |
| τ_00_ Family | 53.65 |  |  |
| τ_00_ Site | 0.82 |  |  |
| ICC | 0.11 |  |  |
| N_Family_ | 8944 |  |  |
| N_Site_ | 22 |  |  |
| Observations | 10788 |  |  |
| Marginal R^2^ | 0.049 |  |  |
| Conditional R^2^ | 0.151 |  |  |
|  |  |  |  |

Full mixed effects model examining the association between family pattern density of mania and delay discounting behavior (area under the curve) in the full sample. The model did not find a significant association between family history of mania and delay discounting behavior when adjusted for socioeconomic and demographic variables.

**Table S5. Mixed Effects Model Examining the Association between Family History of Schizophrenia and Delay Discounting Behavior in the Full Sample with Sociodemographic Factors Included**

| Predictors | Estimates | 95% CI | p |
| --- | --- | --- | --- |
| Family History of Schizophrenia | 0.62 | -3.74, 4.98 | 0.780 |
| **Black** | **-6.81** | **-8.27, -5.35** | **<0.001** |
| Asian | -1.50 | -4.39, 1.40 | 0.310 |
| **Mixed/Other Race** | **-2.12** | **-3.34, -0.89** | **0.001** |
| **Hispanic** | **-3.78** | **-5.17, -2.39** | **<0.001** |
| Age | 0.45 | -0.24, 1.13 | 0.202 |
| **Female** | **2.66** | **1.80, 3.51** | **<0.001** |
| High School Diploma or GED | 0.78 | -1.84, 3.40 | 0.560 |
| Some College | 2.01 | -0.40, 4.41 | 0.102 |
| **Bachelor’s Degree** | **5.11** | **2.56, 7.65** | **<0.001** |
| **Post Graduate Education** | **6.30** | **3.73, 8.88** | **<0.001** |
| Married | 1.46 | 0.31, 2.61 | 0.013 |
| Household Income $50,000 - $99,000 | 1.72 | 0.31, 3.12 | 0.017 |
| Household Income ≥ $100,000 | 1.26 | -0.28, 2.79 | 0.108 |
| Household Income Unknown | 0.76 | -1.06, 2.57 | 0.414 |
| ≥1 Parent Working | -0.86 | -0.80, 2.52 | 0.308 |
| Random Effects |  |  |  |
| σ | 454.03 |  |  |
| τ_00_ Family | 53.93 |  |  |
| τ_00_ Site | 0.79 |  |  |
| ICC | 0.11 |  |  |
| N_Family_ | 8944 |  |  |
| N_Site_ | 22 |  |  |
| Observations | 10788 |  |  |
| Marginal R^2^ | 0.049 |  |  |
| Conditional R^2^ | 0.151 |  |  |
|  |  |  |  |

Full mixed effects model examining the association between family pattern density of schizophrenia and delay discounting behavior (area under the curve) in the full sample. The model did not find a significant association between family history of schizophrenia and delay discounting behavior when adjusted for socioeconomic and demographic variables.

**Table S6. Mixed Effects Model Examining the Association between Family History of Suicidal Behavior and Delay Discounting Behavior in the Full Sample with Sociodemographic Factors Included**

| Predictors | Estimates | 95% CI | p |
| --- | --- | --- | --- |
| Family History of Suicidal Behavior | -0.04 | -2.82, 2.73 | 0.975 |
| **Black** | **-6.81** | **-8.28, -5.35** | **<0.001** |
| Asian | -1.50 | -4.39, 1.39 | 0.310 |
| **Mixed/Other Race** | **-2.11** | **-3.34, -0.89** | **0.001** |
| **Hispanic** | **-3.79** | **-5.18, -2.39** | **<0.001** |
| Age | 0.44 | -0.24, 1.13 | 0.204 |
| **Female Sex** | **2.66** | **1.80, 3.52** | **<0.001** |
| High School Diploma or GED | 0.78 | -1.84, 3.40 | 0.558 |
| Some College | 2.02 | -0.39, 4.42 | 0.100 |
| **Bachelor’s Degree** | **5.11** | **2.57, 7.65** | **<0.001** |
| **Post Graduate Education** | **6.30** | **3.73, 8.88** | **<0.001** |
| Married | 1.45 | 0.31, 2.60 | 0.013 |
| Household Income $50,000 - $99,000 | 1.71 | 0.31, 3.12 | 0.017 |
| Household Income ≥ $100,000 | 1.25 | -0.29, 2.78 | 0.111 |
| Household Income Unknown | 0.75 | -1.07, 2.56 | 0.420 |
| ≥1 Parent Working | 0.85 | -0.81, 2.51 | 0.316 |
| Random Effects |  |  |  |
| σ | 454.00 |  |  |
| τ_00_ Family | 53.97 |  |  |
| τ_00_ Site | 0.79 |  |  |
| ICC | 0.11 |  |  |
| N_Family_ | 8944 |  |  |
| N_Site_ | 22 |  |  |
| Observations | 10788 |  |  |
| Marginal R^2^ | 0.049 |  |  |
| Conditional R^2^ | 0.151 |  |  |
|  |  |  |  |

Full mixed effects model examining the association between family pattern density of suicidal behavior and delay discounting behavior (area under the curve) in the full sample. The model did not find a significant association between family history of suicidal behavior and delay discounting behavior when adjusted for socioeconomic and demographic variables.

**Table S7. Mixed Effects Model Examining the Association between Family History of Alcohol Problems and Delay Discounting Behavior in the Quality Control Sample with Sociodemographic Factors Included**

| Predictors | Estimates | 95% CI | p |
| --- | --- | --- | --- |
| Family History of Alcohol Problems | -1.48 | -3.45 – 0.48 | 0.138 |
| **Black** | **-4.63** | **-7.00 – -2.26** | **<0.001** |
| Asian | -0.81 | -4.63 – 3.00 | 0.676 |
| Mixed/Other Race | -1.32 | -3.08 – 0.43 | 0.139 |
| **Hispanic** | **-3.64** | **-5.65 – -1.63** | **<0.001** |
| Age | 0.49 | -0.48 – 1.45 | 0.323 |
| **Female** | **2.87** | **1.66 – 4.08** | **<0.001** |
| High School Diploma or GED | -0.52 | -5.37 – 4.32 | 0.832 |
| Some College | 2.53 | -1.89 – 6.94 | 0.262 |
| **Bachelor’s Degree** | **5.54** | **1.03 – 10.05** | **0.016** |
| **Post Graduate Education** | **5.97** | **1.43 – 10.51** | **0.010** |
| **Married** | **1.83** | **0.17 – 3.50** | **0.031** |
| Household Income $50,000 - $99,000 | -0.02 | -2.12 – 2.07 | 0.983 |
| Household Income ≥ $100,000 | 0.13 | -2.10 – 2.36 | 0.910 |
| Household Income Unknown | -0.72 | -3.51 – 2.07 | 0.613 |
| ≥1 Parent Working | -0.92 | -3.70 – 1.86 | 0.514 |
| Random Effects |  |  |  |
| σ | 348.79 |  |  |
| τ_00_ Family | 51.12 |  |  |
| τ_00_ Site | 1.24 |  |  |
| ICC | 0.13 |  |  |
| N_Family_ | 3960 |  |  |
| N_Site_ | 22 |  |  |
| Observations | 4288 |  |  |
| Marginal R^2^ | 0.037 |  |  |
| Conditional R^2^ | 0.163 |  |  |
|  |  |  |  |

Full mixed effects model examining the association between family pattern density of alcohol problems and delay discounting behavior (area under the curve) in the sample meeting data quality criteria for the delay discounting task (N=4364). The model did not find a significant association between family history of alcohol problems and delay discounting behavior when adjusted for socioeconomic and demographic variables and applying Bonferroni correction for six comparisons.

**Table S8. Mixed Effects Model Examining the Association between Family History of Drug Problems and Delay Discounting Behavior in the Quality Control Sample with Sociodemographic Factors Included**

| Predictors | Estimates | 95% CI | p |
| --- | --- | --- | --- |
| Family History of Drug Problems | 0.64 | -1.92 – 3.19 | 0.624 |
| **Black** | **-4.43** | **-6.79 – -2.06** | **<0.001** |
| Asian | -0.64 | -4.45 – 3.18 | 0.743 |
| **Mixed/Other Race** | -1.36 | -3.11 – 0.40 | 0.130 |
| **Hispanic** | **-3.54** | **-5.55 – -1.52** | **0.001** |
| Age | 0.50 | -0.47 – 1.46 | 0.313 |
| **Female** | 2.88 | 1.67 – 4.09 | **<0.001** |
| High School Diploma or GED | -0.64 | -5.49 – 4.21 | 0.797 |
| Some College | 2.38 | -2.03 – 6.79 | 0.290 |
| **Bachelor’s Degree** | **5.53** | **1.01 – 10.04** | **0.016** |
| **Post Graduate Education** | **6.01** | **1.47 – 10.56** | **0.009** |
| **Married** | **2.04** | **0.37 – 3.71** | **0.017** |
| Household Income $50,000 - $99,000 | -0.00 | -2.10 – 2.09 | 0.997 |
| Household Income ≥ $100,000 | 0.23 | -2.00 – 2.47 | 0.838 |
| Household Income Unknown | -0.56 | -3.35 – 2.23 | 0.695 |
| ≥ 1 Parent Working | -0.96 | -3.74 – 1.82 | 0.49 |
| Random Effects |  |  |  |
| σ | 349.84 |  |  |
| τ_00_ Family | 50.18 |  |  |
| τ_00_ Site | 1.33 |  |  |
| ICC | 0.13 |  |  |
| N_Family_ | 3960 |  |  |
| N_Site_ | 22 |  |  |
| Observations | 4288 |  |  |
| Marginal R^2^ | 0.037 |  |  |
| Conditional R^2^ | 0.160 |  |  |
|  |  |  |  |

Full mixed effects model examining the association between family pattern density of drug problems and delay discounting behavior (area under the curve) in the sample meeting data quality criteria for the delay discounting task (N=4364). The model did not find a significant association between family history of drug problems and delay discounting behavior when adjusted for socioeconomic and demographic variables.

**Table S9. Mixed Effects Model Examining the Association between Family History of Depression and Delay Discounting Behavior in the Quality Control Sample with Sociodemographic Factors Included**

| Predictors | Estimates | 95% CI | p |
| --- | --- | --- | --- |
| Family History of Depression | 0.67 | -0.62 – 1.97 | 0.308 |
| **Black** | **-4.34** | **-6.71 – -1.96** | **<0.001** |
| Asian | -0.52 | -4.34 – 3.31 | 0.790 |
| Mixed/Other Race | -1.34 | -3.09 – 0.42 | 0.135 |
| **Hispanic** | **-3.49** | **-5.51 – -1.47** | **0.001** |
| Age | 0.50 | -0.46 – 1.47 | 0.304 |
| **Female Sex** | **2.89** | **1.67 – 4.10** | **<0.001** |
| High School Diploma or GED | -0.60 | -5.45 – 4.25 | 0.808 |
| Some College | 2.33 | -2.08 – 6.74 | 0.301 |
| **Bachelor’s Degree** | **5.47** | **0.96 – 9.98** | **0.018** |
| **Post Graduate Education** | **5.93** | **1.39 – 10.47** | **0.011** |
| **Married** | **2.03** | **0.37 – 3.69** | **0.016** |
| Household Income $50,000 - $99,000 | 0.01 | -2.09 – 2.10 | 0.995 |
| Household Income ≥ $100,000 | 0.26 | -1.97 – 2.50 | 0.817 |
| Household Income Unknown | -0.51 | -3.30 – 2.28 | 0.720 |
| ≥Parent Working | -0.95 | -3.73 – 1.83 | 0.50 |
| Random Effects |  |  |  |
| σ | 349.00 |  |  |
| τ_00_ Family | 51.00 |  |  |
| τ_00_ Site | 1.27 |  |  |
| ICC | 0.13 |  |  |
| N_Family_ | 3960 |  |  |
| N_Site_ | 22 |  |  |
| Observations | 4288 |  |  |
| Marginal R^2^ | 0.037 |  |  |
| Conditional R^2^ | 0.162 |  |  |
|  |  |  |  |

Full mixed effects model examining the association between family pattern density of depression and delay discounting behavior (area under the curve) in the sample meeting data quality criteria (N=4364). The model did not find a significant association between family history of depression and delay discounting behavior when adjusted for socioeconomic and demographic variables.

**Table S10. Mixed Effects Model Examining the Association between Family History of Mania and Delay Discounting Behavior in the Quality Control Sample with Sociodemographic Factors Included**

| Predictors | Estimates | 95% CI | p |
| --- | --- | --- | --- |
| Family History of Mania | -1.36 | -5.50 – 2.77 | 0.518 |
| **Black** | **-4.50** | **-6.87 – -2.13** | **<0.001** |
| Asian | -0.71 | -4.52 – 3.11 | 0.717 |
| Mixed/Other Race | -1.34 | -3.09 – 0.42 | 0.136 |
| **Hispanic** | **-3.59** | **-5.61 – -1.58** | **<0.001** |
| Age | 0.49 | -0.47 – 1.46 | 0.315 |
| **Female** | **2.88** | **1.67 – 4.09** | **<0.001** |
| High School Diploma or GED | -0.60 | -5.45 – 4.24 | 0.807 |
| Some College | 2.47 | -1.94 – 6.88 | 0.273 |
| **Bachelor’s Degree** | **5.54** | **1.03 – 10.05** | **0.016** |
| **Post Graduate Education** | **6.01** | **1.46 – 10.55** | **0.010** |
| **Married** | **1.94** | **0.29 – 3.60** | **0.021** |
| Household Income $50,000 - $99,000 | -0.02 | -2.12 – 2.07 | 0.983 |
| Household Income ≥ $100,000 | 0.18 | -2.05 – 2.41 | 0.876 |
| Household Income Unknown | -0.60 | -3.38 – 2.19 | 0.674 |
| ≥ 1 Parent Working | -0.97 | -3.75 – 1.81 | 0.49 |
| Random Effects |  |  |  |
| σ | 349.61 |  |  |
| τ_00_ Family | 50.39 |  |  |
| τ_00_ Site | 1.34 |  |  |
| ICC | 0.13 |  |  |
| N_Family_ | 3960 |  |  |
| N_Site_ | 22 |  |  |
| Observations | 4288 |  |  |
| Marginal R^2^ | 0.037 |  |  |
| Conditional R^2^ | 0.161 |  |  |
|  |  |  |  |

Full mixed effects model examining the association between family pattern density of mania and delay discounting behavior (area under the curve) in the sample meeting data quality criteria (N=4364). The model did not find a significant association between family history of mania and delay discounting behavior when adjusted for socioeconomic and demographic variables.

**Table S11. Mixed Effects Model Examining the Association between Family History of Schizophrenia and Delay Discounting Behavior in the Quality Control Sample with Sociodemographic Factors Included**

| Predictors | Estimates | 95% CI | p |
| --- | --- | --- | --- |
| Family History of Schizophrenia | 3.68 | -3.26 – 10.61 | 0.299 |
| **Black** | -4.48 | -6.84 – -2.12 | **<0.001** |
| Asian | -0.68 | -4.49 – 3.14 | 0.728 |
| **Mixed/Other Race** | -1.37 | -3.13 – 0.38 | 0.126 |
| **Hispanic** | -3.54 | -5.55 – -1.52 | **0.001** |
| Age | 0.49 | -0.47 – 1.46 | 0.314 |
| **Female** | 2.88 | 1.67 – 4.10 | **<0.001** |
| High School Diploma or GED | -0.61 | -5.45 – 4.24 | 0.807 |
| Some College | 2.40 | -2.01 – 6.81 | 0.286 |
| **Bachelor’s Degree** | 5.51 | 1.00 – 10.02 | **0.017** |
| **Post Graduate Education** | 5.99 | 1.45 – 10.53 | **0.010** |
| Married | 2.01 | 0.36 – 3.67 | **0.017** |
| Household Income $50,000 - $99,000 | -0.02 | -2.11 – 2.08 | 0.988 |
| Household Income ≥ $100,000 | 0.23 | -2.00 – 2.46 | 0.842 |
| Household Income Unknown | -0.56 | -3.34 – 2.23 | 0.694 |
| ≥1 Parent Working | -0.96 | -3.74 – 1.82 | 0.497 |
| Random Effects |  |  |  |
| σ | 349.68 |  |  |
| τ_00_ Family | 50.28 |  |  |
| τ_00_ Site | 1.30 |  |  |
| ICC | 0.13 |  |  |
| N_Family_ | 3960 |  |  |
| N_Site_ | 22 |  |  |
| Observations | 4288 |  |  |
| Marginal R^2^ | 0.037 |  |  |
| Conditional R^2^ | 0.161 |  |  |
|  |  |  |  |

Full mixed effects model examining the association between family pattern density of schizophrenia and delay discounting behavior (area under the curve) in the sample meeting data quality criteria (N=4364). The model did not find a significant association between family history of schizophrenia and delay discounting behavior when adjusted for socioeconomic and demographic variables.

**Table S12. Mixed Effects Model Examining the Association between Family History of Suicidal Behavior and Delay Discounting Behavior in the Quality Control Sample with Sociodemographic Factors Included**

| Predictors | Estimates | 95% CI | p |
| --- | --- | --- | --- |
| Family History of Suicidal Behavior | 0.13 | -3.92 – 4.18 | 0.950 |
| **Black** | -4.46 | -6.82 – -2.09 | **<0.001** |
| Asian | -0.67 | -4.48 – 3.15 | 0.732 |
| **Mixed/Other Race** | -1.35 | -3.10 – 0.41 | 0.133 |
| **Hispanic** | -3.57 | -5.58 – -1.55 | **0.001** |
| Age | 0.49 | -0.47 – 1.46 | 0.315 |
| **Female Sex** | 2.88 | 1.67 – 4.09 | **<0.001** |
| High School Diploma or GED | -0.61 | -5.45 – 4.24 | 0.806 |
| Some College | 2.42 | -1.99 – 6.83 | 0.283 |
| **Bachelor’s Degree** | 5.51 | 1.00 – 10.02 | **0.017** |
| **Post Graduate Education** | 5.99 | 1.44 – 10.53 | **0.010** |
| Married | 1.98 | 0.32 – 3.64 | **0.019** |
| Household Income $50,000 - $99,000 | -0.01 | -2.11 – 2.08 | 0.989 |
| Household Income ≥ $100,000 | 0.20 | -2.03 – 2.43 | 0.860 |
| Household Income Unknown | -0.59 | -3.38 – 2.20 | 0.679 |
| ≥1 Parent Working | -0.95 | -3.73 – 1.83 | 0.504 |
| Random Effects |  |  |  |
| σ | 349.53 |  |  |
| τ_00_ Family | 50.53 |  |  |
| τ_00_ Site | 1.32 |  |  |
| ICC | 0.13 |  |  |
| N_Family_ | 3960 |  |  |
| N_Site_ | 22 |  |  |
| Observations | 4288 |  |  |
| Marginal R^2^ | 0.037 |  |  |
| Conditional R^2^ | 0.161 |  |  |
|  |  |  |  |

Full mixed effects model examining the association between family pattern density of suicidal behavior and delay discounting behavior (area under the curve) in sample meeting data quality criteria (N=4364). The model did not find a significant association between family history of suicidal behavior and delay discounting behavior when adjusted for socioeconomic and demographic variables.
